# Herpes Simplex Virus-Associated Aplastic Anemia

**DOI:** 10.7759/cureus.35320

**Published:** 2023-02-22

**Authors:** Oscar A Hinojosa, Omar Ammari

**Affiliations:** 1 Internal Medicine, Henry Ford Health System, Detroit, USA

**Keywords:** herpes simplex virus type 1, viral infection-associated aplastic anemia, viral mucositis, oral mucositis, acquired aplastic anemia

## Abstract

Aplastic anemia is an uncommon condition defined by peripheral pancytopenia in the context of hypocellular bone marrow. In the majority of cases, it is idiopathic in origin. However, exposure to certain drugs and toxins, autoimmune processes, and viral infections have been linked to this entity. This is the case of a 56-year-old female with an acute presentation of fever, odynophagia, and dysphagia. Physical examination revealed multiple hemorrhagic ulcers affecting her oropharyngeal mucosa with regions of necrosis. Mucosal biopsy was compatible with the presence of local necrosis and keratinization. Hematological analysis showed severe peripheral pancytopenia, and the bone marrow biopsy revealed a hypocellular marrow, findings consistent with aplastic anemia. An ample PCR viral panel revealed the presence of herpes simplex virus type 1 (HSV-1). The patient was placed on systemic antiviral therapy, followed by a rapid improvement of the mucositis as well as the peripheral and central pancytopenia. Our case indicated the possible association of HSV-1 infection and the development of aplastic anemia, an important and not yet recognized association considering the rapid improvement of the clinical picture once the underlying etiology was addressed.

## Introduction

Aplastic anemia is an entity characterized by pancytopenia in the setting of hypocellular bone marrow. Its incidence is estimated at 2.2 cases per 1,000,000 persons per year in the International Aplastic Anemia and Agranulocytosis Study, based on European and Israeli populations [1]. While multiple physiopathologic mechanisms are postulated, a common feature is the destruction of the hematopoietic stem cells (HSCs) of the bone marrow [2]. Multiple conditions have been linked to aplastic anemia, including drug exposures, autoimmune disorders, and viral infections. Of the viruses associated with aplastic anemia, the most notable ones are the hepatitis viruses, parvovirus, and human immunodeficiency virus (HIV) [3-5], but no published cases of herpes simplex virus type 1 (HSV-1) were found in the current medical literature.

## Case presentation

**Figure 1:**
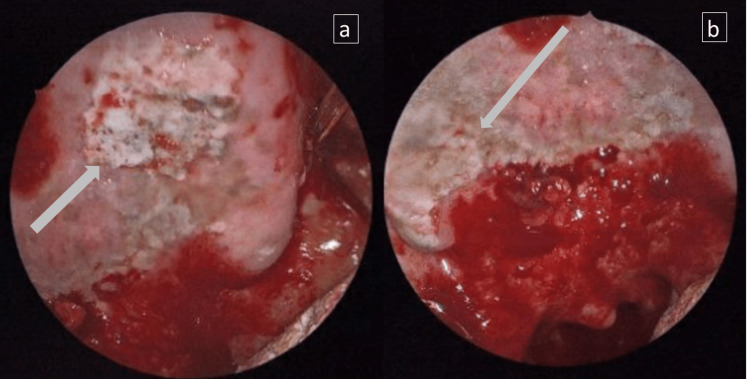
Oropharyngeal endoscopy findings Multiple ulcers in the anterior (a) and posterior (b) palate with necrotic areas (gray arrows)

A 56-year-old African American female with a history of end-stage renal disease (ESRD) on hemodialysis for several years, hypertension, and diabetes presented with a three-day history of odynophagia, dysphagia, fever (101.3°F/38.5°C), and blood-tinged sputum. On physical examination, she was found to have severe mucositis, characterized by significant edema and bleeding ulcers affecting her oral mucosa, tongue, tonsils, and oropharynx. An oropharyngeal endoscopy revealed extensive desquamation and erythema of the buccal and soft palate mucosa, petechiae, and multiple, bilateral, diffuse, gray plaques of necrotic appearance in the hard palate (Figure 1). Multiple biopsies were taken from the hard palate and oropharynx.

The initial workup revealed pancytopenia: hemoglobin (Hgb), 8.9 g/dL; white blood cell (WBC), 1.1 K/uL, with an absolute neutrophil count (ANC) of 0.53 K/uL; and platelets, 84 K/uL. The absolute reticulocyte count was 4-6 K/uL with a reticulocyte index (RTI) of 0.04, indicating a hypoproliferative state. Within 72 hours of the initial presentation, the blood cell count would reach its nadir (Table 1).

**Table 1:** Abnormal hematological laboratory results The baseline is established from a complete blood cell count two months prior to the current presentation. Hgb, hemoglobin; WBC, white blood cell; ANC, absolute neutrophil count

Pertinent hematological laboratory results			
Laboratory results	Baseline	On presentation	Nadir
Hgb (reference range: 12-15 g/dL)	9.4 g/dL	8.9 g/dL	6.6 g/dL
WBC count (reference range: 3.8-10.6 K/uL)	6.7 K/uL	1.1 K/uL	0.4 K/uL
ANC (reference range: 1.8-7.7 K/uL)	4.8 K/uL	0.53 K/uL	0 K/uL
Platelets (reference range: 150-450 K/uL)	274 K/uL	84 K/uL	23 K/uL
Absolute reticulocyte count (reference range: 20.7-83.2 K/uL)	Not available	4.1 K/uL	0 K/uL

The initial frozen section biopsy was concerning for features of a possible invasive fungal infection, which was eventually ruled out on further microscopic analysis by the pathology department. A bilateral nasal endoscopy did not show any significant findings. The patient met the criteria for febrile neutropenia and was placed on broad-spectrum antibiotics, including piperacillin/tazobactam and vancomycin. Due to the concern for *Mucor* infection, amphotericin B was also added to the regimen. Despite these measures, the pancytopenia and stomatitis worsened, requiring multiple transfusions.

Blood and final tissue aerobic, anaerobic, and fungal cultures were negative. The bacterial and viral panel (Table 2) revealed the presence of herpes virus type 1 (HSV-1) in a sample obtained from the oral mucosa.

**Table 2:** Viral and bacterial panel CMV, cytomegalovirus; HBsAg, hepatitis B surface antigen; anti-HBc, hepatitis B core antibody; HCV, hepatitis C virus; HIV, human immunodeficiency virus; HSV-1, herpes simplex virus type 1; HSV-2, herpes simplex virus type 2; NAAT, nucleic acid amplification test; RSV, respiratory syncytial virus

Agent tested	Sample source	Result
HSV-1 NAAT	Oral cavity	Detected
HSV-2 NAAT	Oral cavity	Not detected
Parvovirus B-19 serology for IgM and IgG antibodies	Serum	Not detected
HIV fourth-generation antigen/antibody combo	Serum	Not detected
CMV NAAT	Serum	Not detected
RSV NAAT	Nasopharynx	Not detected
HCV serology for antibodies	Serum	Not detected
HBsAg	Serum	Not detected
Total anti-HBc	Serum	Not detected
Influenza A and B NAAT	Nasopharynx	Not detected
Parainfluenza viruses 1-4 NAAT	Nasopharynx	Not detected
Adenovirus NAAT	Nasopharynx	Not detected
2019-novel coronavirus NAAT	Nasopharynx	Not detected
*Chlamydia pneumoniae* NAAT	Nasopharynx	Not detected
*Mycoplasma pneumoniae* NAAT	Nasopharynx	Not detected

**Figure 2:**
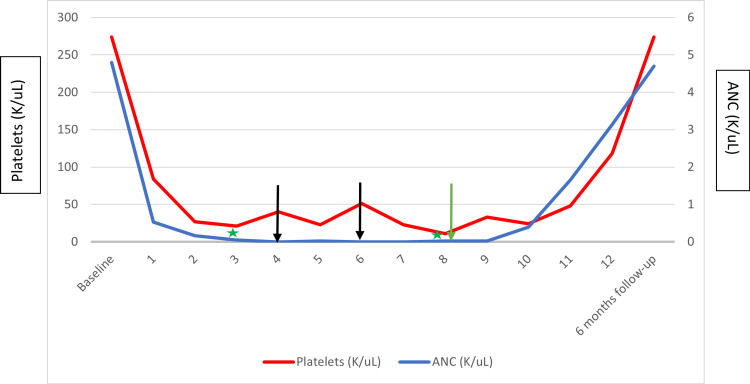
Evolution of platelet and ANC over time Green stars, bone marrow biopsies; black arrows, transfusions of one unit of red blood cells and one unit of platelets; green arrow, beginning of acyclovir therapy ANC, absolute neutrophil count

Due to the worsening pancytopenia, further workup was done. A peripheral smear revealed marked anemia, neutropenia, and thrombocytopenia, without remarkable changes in morphology. Due to the suggestion of hypoproliferation based on the RTI and the peripheral smear findings, a bone marrow biopsy was obtained, which revealed a hypocellular bone marrow (<5% cellularity) without dysplastic or lymphoproliferative features, as well as a normal XX karyotype and no chromosomal or immunophenotypic abnormalities, suggestive of lymphoproliferation. A fluorescent in situ hybridization (FISH) test was negative for mutations on the retinoic acid receptor alpha (*RARA*) and promyelocytic leukemia protein (*PML*) genes. A next-generation sequencing assay was negative for mutations on the following genes: colony-stimulating factor 3 receptor (*CSF3R*), neuroblastoma rat sarcoma virus (*NRAS*), CCAAT box enhancer-binding protein alpha (*CEBPA*), deoxyribonucleic acid (cytosine-5)-methyltransferase 3A (*DNMT3A*), runt-related transcription factor 1 (*RUNX1*), GATA box binding protein 2 (*GATA2*), cut-like homeobox 1 (*CUX1*), enhancer of zeste homolog 2 (*EZH2*), B-cell lymphoma corepressor (*BCOR*), stromal antigen 2 (*STAG2*), and BCOR ligand 1 (*BCORL1*). The bone marrow was also negative for HSV on the immunohistochemical stain. Based on the findings of HSV-1 on the oral mucosa, an acyclovir regimen (dose of 5 mg/kg) was administered for seven days, resulting in rapid symptomatic improvement and recovery of all cell lines as displayed in Figure 2.

A second bone marrow biopsy confirmed the rapid recovery in cellularity (20%-30%) with unchanged immunophenotypic, chromosomal, and genetic profiles.

The patient did not have any known exposure to radiation or associated anti-inflammatory, antimicrobial, anticonvulsant, or chemotherapeutic agents. No heavy metal levels were measured. The patient had no history of autoimmune/connective tissue disorders. An autoimmune panel revealed the presence of antinuclear antibodies (ANA) on a homogeneous pattern, with a 1:160 titer. The rest of the workup was noncontributory (Table 3).

**Table 3:** Summary of laboratory findings ANA, antinuclear antibody; C3, complement component 3; C4, complement component 4; C-ANCA, cytoplasmic antineutrophil cytoplasmic antibody; DNA Ab (ds), anti-double-stranded deoxyribonucleic acid antibody; P-ANCA, perinuclear antineutrophil cytoplasmic antibody

Parameter	Value	Normal range
ANA titer	1:160	Titer < 1:80
C-ANCA	<1:20	Titer < 1:20
P-ANCA	<1:20	Titer < 1:20
DNA Ab (ds)	Not detected	<10 UI/mL
Centromere antibody	Not detected	Titer < 1:180
C3 complement	129	90-230 mg/dL
C4 complement	44	10-51 mg/dL
Vitamin B12	887	180-810 pg/mL
Folate	15.9	> 6.6 ng/mL

Based on the rapid recovery of the cell counts in the peripheral blood as well as in the bone marrow biopsy samples, with normal cytogenetics, after the antiviral treatment was started, the patient was diagnosed with aplastic anemia likely provoked by HSV-1 infection. Although the patient had an elevated ANA titer, an autoimmune condition was deemed less likely based on the absence of other features of autoimmune conditions and the fact that the stomatitis and pancytopenia improved without corticosteroid or immunosuppressant administration.

## Discussion

Aplastic anemia is a rare condition [1] defined by the presence of peripheral cytopenias and bone marrow hypocellularity (<30%), which can not be attributed to dysplasias, lymphoproliferative disorders, or bone marrow fibrosis [2]. The exact physiopathologic mechanism behind it is unknown, and in most cases, an exact etiology is never identified, but multiple theories have been postulated. Bone marrow hypocellularity can be attributed to direct damage to the bone marrow by chemotherapy, radiation, or toxin exposure (benzene, organophosphates, sulfonamides, aspirin, colchicine, hydroxychloroquine, allopurinol, phenytoin, carbamazepine, diclofenac, etc.); this mechanism is usually dose-dependent. It can also be the product of constitutional syndromes such as Fanconi anemia or dyskeratosis congenita. In these cases, there are germline mutations that impair the capacity of hematopoietic stem cells (HSCs) in the bone marrow to repair their own deoxyribonucleic acid (DNA); in other cases, the mutations can affect cells in charge of immunity regulation. Since most of these mutations are inherited, they classically manifest during childhood [2].

Finally, bone marrow hypocellularity can also be attributed to an autoimmune process. As a response to drug exposure, viral infection, or other conditions, there is T-cell-mediated HSC destruction, resulting in subsequent peripheral pancytopenia [2,3]. This last mechanism could potentially explain the absence of a viral agent in our patient’s bone marrow biopsy. This mechanism is related to immunologic disorders (rheumatoid arthritis and lupus) and pregnancy [2], as well as viral infections [3]. The viruses more commonly associated with aplastic anemia include hepatitides viruses, parvovirus, and HIV [4,5]. Although there are limited case reports associating aplastic anemia with seronegative hepatitis, varicella-zoster virus (VZV), Epstein-Barr virus (EBV), and HSV-6 infections [4,6-11], no reported cases of HSV-1-associated aplastic anemia were identified in the current medical literature.

Based on the severity of the cytopenias, the condition can be classified as moderate, severe, or very severe. Our patient fulfilled all the criteria for very severe aplastic anemia (Hgb < 8 g/dL, reticulocyte count < 20 K/uL, ANC < 0.2 K/uL, and platelets < 20 K/uL) [12]. Regarding management, mild and moderate cases are followed with active observation. Most of the adults that fall in the category of severe or very severe aplastic anemia will require immunosuppressive therapy in the form of antithymocyte globulin, usually in combination with cyclosporine, or if the patient is <40 years, the patient may qualify for an allogeneic bone marrow transplantation [2,12].

## Conclusions

Internists that face a clinical scenario of rapidly developing pancytopenia compatible with aplastic anemia should consider a viral infection as the possible etiology. Most cases of severe aplastic anemia will require immunosuppressive therapy or bone marrow transplantation. However, if a treatable entity is identified, such as HSV-1, its management may hasten the recovery and avoid further escalation of treatment.
